# A database of marine larval fish assemblages in Australian temperate and subtropical waters

**DOI:** 10.1038/sdata.2018.207

**Published:** 2018-10-16

**Authors:** James A. Smith, Anthony G. Miskiewicz, Lynnath E. Beckley, Jason D. Everett, Valquíria Garcia, Charles A. Gray, David Holliday, Alan R. Jordan, John Keane, Ana Lara-Lopez, Jeffrey M. Leis, Paloma A. Matis, Barbara A. Muhling, Francisco J. Neira, Anthony J. Richardson, Kimberley A. Smith, Kerrie M. Swadling, Augy Syahailatua, Matthew D. Taylor, Paul D. van Ruth, Tim M. Ward, Iain M. Suthers

**Affiliations:** 1 School of Biological, Earth, and Environmental Sciences, University of New South Wales, Sydney, NSW 2052, Australia; 2 Sydney Institute of Marine Science, Mosman, NSW 2088, Australia; 3 Environment and Strategic Planning, Wollongong City Council, PO Box 8821, Wollongong, NSW 2500, Australia; 4 Environmental and Conservation Sciences, School of Veterinary and Life Sciences, Murdoch University, Murdoch, WA 6150, Australia; 5 Ichthyology, Australian Museum Research Institute, Sydney, NSW 2010, Australia; 6 Laboratório de Biologia e Cultivo de Peixes de Água Doce, Programa de Pós-Graduação em Aquicultura, Universidade Federal de Santa Catarina, Florianópolis, 88040-900, Brazil; 7 Wildfish Research, Grays Point, NSW 2232, Australia; 8 NSW Department of Primary Industries, PO Box 4297, Coffs Harbour, NSW 2450, Australia; 9 Institute of Marine and Antarctic Studies, University of Tasmania, Hobart, TAS 7001, Australia; 10 School of Biological Sciences, University of Sydney, Sydney, NSW 2006, Australia; 11 NOAA Southwest Fisheries Science Centre, La Jolla, CA 92037, USA; 12 University of California Santa Cruz, Cooperative Institute for Marine Ecosystems and Climate (CIMEC), La Jolla, CA 92037, USA; 13 Marscco Marine Sciences Consulting, Blackmans Bay, TAS 7052, Australia; 14 CSIRO Marine and Atmospheric Research, Ecosciences Precinct, Brisbane, QLD 4102, Australia; 15 Centre for Applications in Natural Resource Mathematics, School of Mathematics and Physics, The University of Queensland, St Lucia, QLD 4072, Australia; 16 Department of Primary Industries and Regional Development, 39 Northside Drive, Hillarys, WA 6025, Australia; 17 Antarctic Climate and Ecosystems Cooperative Research Centre, Private Bag 80, Hobart, TAS 7001, Australia; 18 Centre for Deep-Sea Research, Indonesian Institute of Sciences, JI. Y. Syaranamual, Poka, Ambon 97233, Indonesia; 19 NSW Department of Primary Industries, Port Stephens Fisheries Institute, Locked Bag 1, Nelson Bay, NSW 2315, Australia; 20 South Australian Research and Development Institute (Aquatic Sciences), PO Box 120, Henley Beach, Adelaide, SA 5022, Australia

**Keywords:** Marine biology, Ichthyology, Community ecology, Biodiversity

## Abstract

Larval fishes are a useful metric of marine ecosystem state and change, as well as species-specific patterns in phenology. The high level of taxonomic expertise required to identify larval fishes to species level, and the considerable effort required to collect samples, make these data very valuable. Here we collate 3178 samples of larval fish assemblages, from 12 research projects from 1983-present, from temperate and subtropical Australian pelagic waters. This forms a benchmark for the larval fish assemblage for the region, and includes recent monitoring of larval fishes at coastal oceanographic reference stations. Comparing larval fishes among projects can be problematic due to differences in taxonomic resolution, and identifying all taxa to species is challenging, so this study reports a standard taxonomic resolution (of 218 taxa) for this region to help guide future research. This larval fish database serves as a data repository for surveys of larval fish assemblages in the region, and can contribute to analysis of climate-driven changes in the location and timing of the spawning of marine fishes.

## Background & Summary

The early life history of most marine fishes occurs in the upper water column, with eggs and larvae developing as part of the plankton, before leaving the plankton for settlement as post-larvae. Surveys of larval fishes (which, together with fish eggs, are termed ichthyoplankton) are one tool for monitoring marine ecosystems and fish communities^1^. Surveys of larval fishes are valuable for ecosystem monitoring because many oceanographic, biological, and anthropogenic processes influence their distribution, abundance, and survival^2–6^. Survey data has been used for monitoring spawning habitats^7,8^, and changes in phenology^9^ and the spawning biomass of adult populations^10^, and may be useful in this Australian region for helping interpret ecosystem changes in a climate change hotspot^11,12^ undergoing substantial biological changes^13,14^.

**Figure 1:**
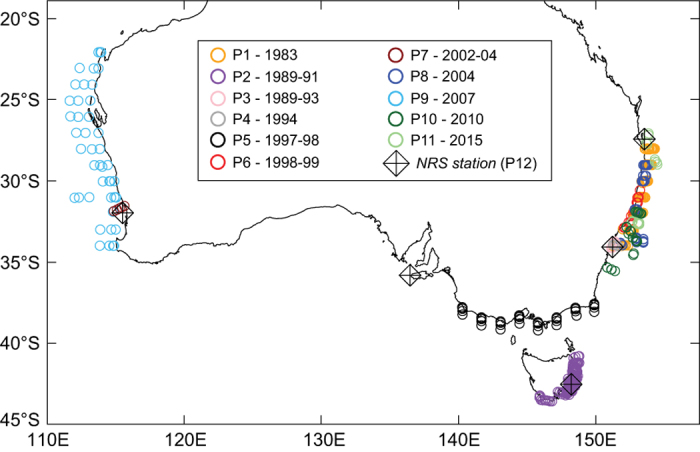
Locations of data for each project (P1-P12) in this data descriptor. See Table 1 for project descriptions. The five National Reference Stations (NRS) at which ~monthly sampling of larval fishes is ongoing (the NIMO program) are indicated. Samples at the three NRS on the east coast are currently being sorted and identified, with the remainder stored for future sorting and identification.

Ichthyoplankton have been surveyed in Australia since the early 20th century, with a 1910 survey of three species in Port Phillip Bay^15^, and surveys in the 1930–50 s of larvae and eggs of sardine (*Sardinops sagax*) and anchovy (*Engraulis australis*)^16–18^. Descriptions of larvae for various species occurred from the 1950s onwards, and in the 1980s surveys of larval fish assemblages began in earnest in Australian temperate marine waters (reviewed in^19^). Since 2014, larval fishes have been routinely collected at five reference stations (Fig. 1), with samples sorted and larval fishes identified at the three east coast stations. Abundances of larval fishes from numerous research voyages were collated in a 2002 report on ~45 commercially-important fish species^20^, with the data provided on a compact disc. There have otherwise been no attempts at collating and sharing larval fish assemblage data from temperate and subtropical Australia.

One challenge with collating larval fish data is a difference in taxonomic resolution among studies, which limits the ability to make robust comparisons of larval fish assemblages. Few scientists have the ability to identify many taxa to species level, and given the progressive loss of taxonomic expertise (and that a large proportion of fish species are endemic to southern Australia)^21^, there is some uncertainty as to whether this expertise will extend to a new generation of marine scientists^22^. This suggests that the taxonomic resolution of future surveys of larval fishes could decline without some guidance. DNA barcoding methods can greatly enhance the identification of ichthyoplankton^23,24^, and potentially reduce the reliance on taxonomic experts; but for generating larval fish count data from large surveys, DNA methods are currently complimentary to morphological identification methods.

This study had two aims: 1) to collate marine larval fish assemblage data from the 1980s onwards for temperate and subtropical Australian waters; and 2) to create a standard taxonomic resolution for these data, which can act as a target resolution for future larval fish research in the region (when identifying all taxa to species is not feasible). The data collated here have come from research voyages (1983-2015), as well as current monitoring at three coastal reference stations (2014 onwards; Table 1, Fig. 1). It consists of 3178 samples and >490,000 identifications. The research voyages were done by a variety of universities and government agencies, and often with environment- or species-specific research objectives: e.g. monitoring the impact of sewage ocean outfalls on larval fishes^25^. These various objectives are reflected in the broad range of spatial and temporal scales of these surveys (Table 1). The recent monitoring data are an initiative under the auspices of the Australian Integrated Marine Observing System (IMOS), which monitors multiple physical and bio-chemical properties at seven National References Stations (NRS). This monitoring initiative has been called ‘NIMO’ (National Ichthyoplankton Monitoring and Observing) and began at some of these NRS in late 2014^26^. Together, data from the collated voyages and recent monitoring create a broad understanding of the larval fish assemblages of temperate and subtropical Australia.

This paper begins a database for Australian larval fish assemblages, which acts as a repository for future larval fish surveys and monitoring in this region. The expert-derived taxonomic resolution used for these data can also act as a guide for a minimum resolution of future surveys, and is aimed at resolving all common, commercially-important, and readily identifiable marine fish taxa in this region, while remaining accessible to taxonomists and sorters beyond the few experts who created the species list used in this database. This database joins only a few data sets^1,27^ in providing regional ichthyoplankton survey data collected over a relatively long period.

This Australian larval fish database will be available through the Australian Ocean Data Network portal (AODN: https://portal.aodn.org.au/), the main repository for marine data in Australia. The Australian larval fish database will be maintained and updated through the Commonwealth Scientific and Industrial Research Organisation (CSIRO) data centre, with periodic updates sent to the AODN. A snapshot of the Australian larval fish database at the time of this publication has been assigned a DOI and will be maintained in perpetuity by the AODN (Data Citation 1).

## Methods

### Larval fish sampling

There are a variety of methods for surveying larval fish in the pelagic environment, including towing nets at specific depths, obliquely across a depth range, or at the surface^28–30^. In this database, all larval fishes were sampled by vessel-towed plankton nets towed obliquely or at constant near-surface depths. Upon net retrieval of a single ‘tow’, all plankton were fixed immediately in ~4% formalin in seawater (and often buffered with sodium borate or sodium carbonate to avoid sample degradation). The volume sampled by the net for each tow was determined, typically using a flowmeter attached to the mouth of the net, which was used to standardise larval fish counts to volume of water sampled. The types of nets used and the depths surveyed varied among studies, and are detailed in Table 1 (refer to key references for each project for more information).

### Larval fish identification

In the laboratory, larval fishes in each sample were sorted, enumerated, and identified. Identification of species was done using key reference guides^19,31–36^ (and others^22^), and frequently through direct contact with experts. For example, AGM or FJN (who co-edited^19^) were involved in the identification of some species in nearly every project in this study. Larval fishes were then stored in ethanol for later reference, and a subset has been archived with the Australian Museum^22^. A list of best reference guides for the identification of each taxon in this study is provided alongside this database (see ‘Data Records’).

### Taxonomic resolution

The taxonomic resolution often varies among projects, which can complicate comparison of larval fish assemblages. An aim of this study was to create a standard taxonomic resolution for surveys of larval fish assemblages in temperate and subtropical Australia. We created a database species list to act as a guide for an ideal minimum resolution for surveys of larval fishes in this Australian region. The goals of this species list were: 1) that it included all common and as many commercially important species as possible; 2) that this taxonomic level could be achieved with a reasonable level of training and reference to existing guides. A working group led by AGM, FJN, JML, and JK, met at the University of Tasmania on 7–9^th^ December 2015, and the resulting species list (with some subsequent revision) is used in this data paper and stored online as associated metadata. The species list consists of a higher order division (usually family), a genus and species (when appropriate), and a common name. Each taxon is also identified with a unique CAAB number. CAAB (Codes for Australian Aquatic Biota) is an 8-digit coding system maintained by CSIRO (http://www.marine.csiro.au/caab/). There are an additional three groups in the species list identified with text: ‘Unknown’, ‘Damaged’, and ‘Other’. To create a single matrix file, each taxon is identified with a single header consisting of ‘Family_Species_CAAB’. If a taxon is only identified to Family, the header is ‘Family_CAAB’. ‘Other’ is used as a ‘Species’ term to indicate when a taxon contains all other species of that family. For example, the taxon ‘Acropomatidae_other_37311956’ includes all species in Acropomatidae except ‘Acropomatidae_Synagrops.spp_37311949’ and ‘Acropomatidae_Apogonops.anomalus_37311053’.

All projects in this study have been aligned to this database species list. Given that each of the projects included here had identification input from the same few experts (AGM, FJN), most taxa could be matched directly. Taxonomic resolutions were occasionally simplified (e.g. multiple species grouped in a single family), and in all cases AGM ensured the alignment was accurate. In rare cases, AGM examined stored samples from specific projects to ensure identifications were accurate.

### Project selection

For this database, we selected a range of projects from temperate and subtropical Australia that surveyed marine pelagic larval fish assemblages and had high taxonomic resolution, and could be aligned to our common database species list with accuracy. This led to 11 projects suitable for inclusion, with the 12^th^ project being the ongoing NIMO monitoring program (Fig. 1, Table 1).

The projects reported in this study are not an exhaustive list of larval fish surveys in Australia. There have been numerous surveys in more tropical areas (e.g.^37–40^); some with species-specific surveys (e.g. sardine, mackerel, blue grenadier^41–44^), and some surveys using vertical hauls (e.g.^4^). These (and others, see^20^) were not included either because they were outside the geographic area of interest, were not at the desired taxonomic resolution, or required investment (in species alignment and quality control) beyond what this study could achieve. However, it is likely that some existing data sets could be added to this database given investment by the data custodians.

### Environmental data

Water temperature and salinity were often collected with each plankton tow, usually measured using a CTD. In some cases these were reported separately and needed to be aligned with the larval fish records. In all cases, except the NRS monitoring and project P3 (Table 1), water temperature of the surface (<10m) waters was available at each tow. Surface salinity for each record was also available for six of the projects.

## Data Records

All data are combined into a single data set, with data distinguished by ‘project’ (Table 1). A project is defined as a set of data records that have been collected together, usually as a voyage or study, and have the same sampling and analysis methods and the same person(s) identifying the larval fishes.

Each record in a project represents a single plankton net tow, with larval fishes reported as counts per tow. Each record has a unique identifier called ‘Tow_ID’ (Table 2), consisting of the Project_ID (Table 1) followed by a consecutive record number within that project. Tow volumes (m^3^) are reported for each record, to allow standardisation to unit volume (per m^3^). Larval fish abundance is sometimes reported per unit area over the sampled depth range (per m^2^) by multiplying by the depth surveyed^5^, and this could be calculated for each record using the information on depths sampled.

Most metadata (e.g. tow depth, water temperature) are provided alongside each record; these record-specific metadata are defined in Table 2. Project-specific metadata (e.g. net type, mesh size) are provided in Table 1, and a non-exhaustive list of published studies are referenced for each project, and can be referred to for further project information (Table 1). Original sample ID codes and survey-specific metadata are used where appropriate to retain traceability with the original data. Missing or non-applicable data are left blank.

Key personnel are listed for each project (Table 1), and these people were involved in data collection and processing, and are usually the original custodians of the samples and data. The database species list to which all data were aligned is also provided as a stand-alone file (https://catalogue-imos.aodn.org.au/geonetwork/srv/en/metadata.show?uuid=2d2b2f92-12fa-4330-a480-94f0892c2b72). Within this file is also a list of best reference texts and guides for the identification of each species in this list, with priority given to references that identify the species, then to references that identify only the genera or family.

## Technical Validation

The original identification of larval fishes in each survey was done with reference to key reference texts (see ‘*Larval fish identification*’ above). Every dataset presented here has been re-examined by AGM to ensure all identifications are within expected spatial and temporal domains. The alignment of taxa to the common species list was done by JAS in consultation with AGM to ensure the alignment was consistent (given revisions in taxonomy).

## Usage Notes

This dataset snapshot is freely available from the following metadata record at (https://catalogue-imos.aodn.org.au/geonetwork/srv/en/metadata.show?uuid=2d2b2f92-12fa-4330-a480-94f0892c2b72). Larval fish monitoring at the IMOS National Reference Stations (NIMO) is ongoing (Fig. 1), and these data will continue to be updated for the duration of the NIMO program. Contact IMOS for status of these data.

Our goal is that the species list in this database remains fixed, but minor updates that do not alter the ability to compare the datasets presented here may occur. Any changes to the species list will be updated on the NRS data files and on the database version of the species list. When data are added to this database, the taxonomic resolution should ideally include all taxa within this species list (to maintain this minimum taxonomic resolution) to enable comparison of all datasets.

## Additional information

**How to cite this article**: Smith, JA *et al.* A database of marine larval fish assemblages in Australian temperate and subtropical waters. *Sci. Data*. 5:180207 doi: 10.1038/sdata.2018.207 (2018).

**Publisher’s note**: Springer Nature remains neutral with regard to jurisdictional claims in published maps and institutional affiliations.

## Supplementary Material



## Figures and Tables

**Table 1:** Description of the historical larval fish surveys used in this report.

**Project ID**	**Project name**	**Years**	**Location (see Fig. 1)**	**Personnel**	**Samples**	**Survey details**	Refs
P1	Sprightly_83	1983	QLD and NSW	AGM	184 (obl.) 184 (surf.)	Vessel: *RV Sprightly*; 3 cruises: Jan. 1983, Mar. 1983, May 1983; includes sampling close to Port Hacking NRS; predominantly off the continental shelf; ring net 500 μm mesh	^45^
P2	Challenger_89	1989-91	Eastern and southern TAS	ARJ	380 (obl.)	Vessel: *RV Challenger*; 3 summer cruises; multiple stations sampled repeatedly; predominantly on the continental shelf; bongo net 500 μm mesh (obl.)	^46^
P3	Kamala_89	1989-93	Sydney, NSW	CAG, AGM	555 (obl.) 543 (surf.)	Vessel: *FRV Kamala*; 14 cruises; repeated measurements at 6 sites and multiple depths close to the coast, monitoring Sydney’s deep ocean sewage outfall; ring net 500 μm mesh	^25,47^
P4	Franklin_94	1994	Sydney, NSW	KS, IMS	147 (obl.) 76 (surf.)	Vessel: *ORV Franklin*; 5 stations sampled repeatedly in Jan 1994 and again in April 1994; both on and off continental shelf; includes sampling close to Port Hacking NRS; EZ net 333 um mesh (obl.), neuston net 500 μm mesh (surf.)	^48,49^
P5	Franklin_97	1997-98	VIC and SA	FJN	318 (obl.) 126 (surf.)	Vessel: *ORV Franklin*; 4 cruises: Jan. 1997, Dec. 1997, June 1998, July 1998; sampled 8 transects repeatedly; 4 depth strata in oblique tows; ~40% of samples were in the 3 eastern most transects (Fig. 1); EZ net and bongo net 500 um mesh (obl.), neuston 500 μm net (surf.)	^50^
P6	Franklin_98	1998-99	NSW	IMS, AS	49 (obl.) 83 (surf.)	Vessel: *ORV Franklin*; Sampled 9 ‘locations’ over 2 cruises: Nov. 1998, Jan. 1999; all on continental shelf; includes sampling close to Port Hacking NRS; EZ net 500 um mesh (obl.), neuston net 500 μm mesh (surf.)	^51–53^
P7	TwoRocks_02	2002-04	Two Rocks, WA	LEB, BAM	91 (obl.)	Vessels: *Maritime Image*, *Mesocat*, *RV Southern Surveyor*; 24 cruises; repeated sampling at 5 stations on a transect (all seasons) as part of the Strategic Research Fund for the Marine Environment (SRFME) program; sampled multiple ocean features; bongo net 355 μm mesh; max tow depth 150 m (obl.)	^54–57^
P8	SouthSurv_04	2004	NSW	IMS	48 (obl.) 60 (surf.)	Vessel: *RV Southern Surveyor*; Single cruise in Sept. 2004; sampled multiple ocean features (EAC, mixed, coast, front); both on and off continental shelf; RMT net 1000 um mesh (obl.), neuston net 500 μm mesh (surf.)	^58^
P9	SouthSurv _07	2007	WA	LEB, DH	96 (obl.)	Vessel: *RV Southern Surveyor*; 13 cross-shelf transects (one per degree latitude); on and off continental shelf; bongo net 355 μm mesh; max tow depth 150 m (obl.)	^6,59,60^
P10	SouthSurv _10	2010	NSW	MDT, PM, IMS	31 (obl.)	Vessel: *RV Southern Surveyor*; Single cruise in Oct. 2010; sampled multiple ocean features; both on and off continental shelf; MOCNESS net 330 μm mesh; top ~ 50 m	^61^
P11	Investigator_15	2015	QLD and NSW	IMS, VG	63 (obl.) 30 (surf.)	Vessel: *RV Investigator*; Single cruise in June 2015; sampled two water types: ‘coast’ and ‘eddy’; both on and off continental shelf; EZ net 500 μm mesh (obl.), neuston net 500 μm mesh (surf.)	
P12_NSI P12_PH P12_MAI	NIMO_NRS	2014 onwards	See Fig. 1	IMS, JAS, AL-L	114 (obl.)	Monthly sampling at three National Reference Stations (NRS) as part of the NIMO program (NSI is North Stradbroke Island; PH is Port Hacking; MAI is Maria Island); ring net 500 μm mesh; surface 10-20 m; sampling and sample processing is ongoing	^26^
Samples are distinguished as surface (surf.) or oblique (obl.) tows. This is a total of 3178 samples. Personnel are those authors involved in key aspects of the survey and/or sample processing, and in most cases are the original custodians of the data.							

**Table 2:** Record-specific metadata provided within the data files and their definitions.

**Project_ID; Project_name**	**Unique database code to identify the project (Table 1)**	**Replicate**	**Identifies when tows were done consecutively to act specifically as replicates**
**Tow_ID**	A unique identifier for each tow, which is the Project_ID plus the numerical data record for that project; e.g. P1_1 is the first record in project P1	**Cruise_ID**	A project-specific identifier of different cruises
**Sample**	A project-specific identifier for each record	**Station**	A project-specific identifier of a specific location sampled multiple times
**Date**	Date a tow was taken, yyyy-mm-dd	**Gear_depth_m**	The maximum depth tows were deployed to, or a range of depths sampled; surface tows have a depth = 0
**Time_local**	The local time a tow was taken, hh24:mm	**Gear_mesh_um**	The type of net, and the mesh dimensions (μm); summarised in Table 1
**Day_Night**	Whether the tow occurred in the day or night – only used when Time_local was unavailable	**Bathym_m**	The bottom depth at location of the tow (m)
**Latitude, Longitude**	Geographic coordinates for each tow (usually start of tow)	**Temperature_C**	Surface (<10 m) water temperature (°C) at approx.. the same time as the tow; measured with a CTD
**Location**	A project-specific identifier of feature of interest (e.g. oceanographic feature)	**Salinity**	Surface (<10 m) salinity (PSU) at approx. the same time as the tow; measured with a CTD
**Volume_m3**	The volume of a tow (m^3^)		
Not all metadata are available/relevant for each project.			
